# Current-reinforced random walks for constructing transport networks

**DOI:** 10.1098/rsif.2012.0864

**Published:** 2013-03-06

**Authors:** Qi Ma, Anders Johansson, Atsushi Tero, Toshiyuki Nakagaki, David J. T. Sumpter

**Affiliations:** 1 Mathematics Department, Uppsala University, Uppsala, Sweden; 2 Mathematics Department, University of Gävle, Gävle, Sweden; 3 PRESTO, JST, 4-1-8 Honcho, Kawaguchi, Saitama, Japan; 4 Faculty of Systems Information Science, Future University Hakodate, Hakodate, Japan

**Keywords:** reinforced random walk, shortest path problem, transport networks, ant algorithm, true slime mould, optimization

## Abstract

Biological systems that build transport networks, such as trail-laying ants and the slime mould *Physarum*, can be described in terms of reinforced random walks. In a reinforced random walk, the route taken by ‘walking’ particles depends on the previous routes of other particles. Here, we present a novel form of random walk in which the flow of particles provides this reinforcement. Starting from an analogy between electrical networks and random walks, we show how to include current reinforcement. We demonstrate that current-reinforcement results in particles converging on the optimal solution of shortest path transport problems, and avoids the self-reinforcing loops seen in standard density-based reinforcement models. We further develop a variant of the model that is biologically realistic, in the sense that the particles can be identified as ants and their measured density corresponds to those observed in maze-solving experiments on Argentine ants. For network formation, we identify the importance of nonlinear current reinforcement in producing networks that optimize both network maintenance and travel times. Other than ant trail formation, these random walks are also closely related to other biological systems, such as blood vessels and neuronal networks, which involve the transport of materials or information. We argue that current reinforcement is likely to be a common mechanism in a range of systems where network construction is observed.

## 1. Introduction

**Figure 1.**
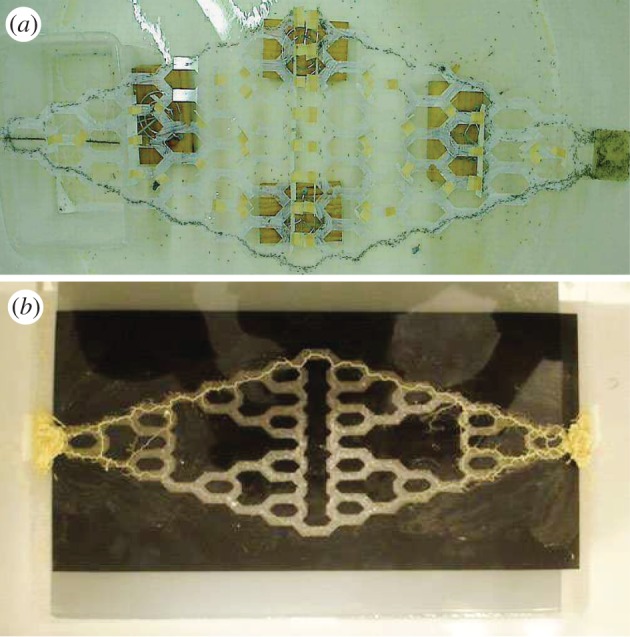
Biological systems solve the ‘Tower of Hanoi’ maze. Each node represents a configuration of a three-rod towers of Hanoi, each edge connects possible move from different states of the discs. See Reid *et al*. [1] for the detailed construction of the network. (*a*) Argentine ants solve the Tower of Hanoi maze. Under the left end of the maze lies the nest, food is placed at the right end of the maze. (*b*) *Physarum* solving the Tower of Hanoi maze. Food sources are placed at both ends of the maze. *Physarum* is evenly spread in the maze at the beginning of the experiment. The figure presents the result 12 h after the start of the experiment. We thank Chris Reid for providing these photographs of his experiments.

Pheromone trail laying and following by ants is a key example of biological problem-solving. As a recent example, Reid *et al.* [1] set up a ‘Towers of Hanoi’ maze for Argentine ants to solve. They put the ant nest at one end of the maze and placed food at the other end. The ants solved this maze by forming a path from their nest to the food source with the shortest length (figure 1*a*). When the shortest path was blocked, they adapted to the new shortest path [1]. Similar examples are seen in the acellular slime mould, *Physarum polycephalum*. *Physarum* circulates nutrients and signals via a network of tubes. Several experiments have shown that *Physarum* can find the shortest path connecting food sources, even in complex mazes [2,3]. Figure 1*b* gives one such example where *Physarum* solves the same Tower of Hanoi maze as the ants. At the beginning of the experiment, the organism was placed through the whole maze, then the food sources were added at either end. Tubes in the longer paths gradually die out and after 12 h, only tubes in the shortest path were left.

Ants and slime moulds solve other combinatorial optimization problems. These include connecting multiple food sources or nests with shortest length [4], solving the Steiner tree problem between three and four food sources/nests [4,5], adapting the shortest path to a minimum-risk path when exposed to a risky environment [3] and building efficient transportation networks [6,7].

How does a biological system without global information solve such problems? In physical appearance, the problem-solving of ants and slime moulds is very different. There are however strong similarities in terms of the underlying algorithm used. There are direct parallels between the growth of tubes in *Physarum* and the creation of pheromone trails in ants. Both involve the random walk of nutrients and ants, respectively. Furthermore, there is a process of reinforcement whereby the more ants/nutrients that pass a particular point the greater the concentration of pheromone/thickness of tubes. In short, they can both be described as reinforced random walks.

Random walks are widespread in biology [8,9], and reinforced random walks are widely used to model aggregation and pattern formation [10–12]. For example, Othmer & Stevens [10] present several possible reinforcement schemes for random walks of particles, with the aim of modelling cell migration. They identify conditions for spatial aggregation of particles, and look at how these depend on interactions between a control substance and the particles that produce this control substance. Similar models have been applied in modelling tumour-induced angiogenesis [13] and to explain the formation of human trail systems [14,15]. In these reinforced random walk models, the control substance is reinforced proportionally to the particles' density [11].

Here, we focus on control substance reinforcement proportional to the particles' gradient. The main difference between our approach and those cited earlier is that we look at current or traffic flow reinforcement. We use an analogy, first proposed by Tero *et al.* [6,16] in the form of the *Physarum* solver, between current reinforcement and tube growth. However, unlike the *Physarum* solver, which is a system of linear equations in which the flow is calculated globally by Kirchoff's law, here we aim to derive a local description of reinforcement in terms of particle motion.

We have several requirements in our derivation of a current-reinforced random walk. First, the particles should change and respond only to their local environment. Second, we should be able to identify the variables in our model with biologically meaningful entities such as ants and pheromone. Third, our model should reproduce the results of the experiments discussed above, both for shortest path problems and for transport networks. We now develop our model in a number of stages. We start §2 by describing an analogy between random walks and electrical networks. We then introduce current reinforcement, where particles reinforce the edges they traverse. Through a series of examples, we develop a biologically realistic description of how ants and *Physarum* implement current reinforcement. This algorithm is shown to converge to the shortest path between two points in a network. In §3, we show how similar algorithms construct optimized networks.

## 2. Shortest path problems

### 2.1. Random walks on networks

First, consider a random walk on a graph in which between two nodes *i* and *j*, there is a resistance proportional to the length of the connection *l_ij_*. A particle enters the network at a source *s* and prefers to move down edges with lower resistance. Specifically, when the particle is on node *i*, with probability2.1
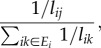
it will move next to node *j*. *E_i_* is the set of edges connected to *i*. The particle continues to chose its travel path in this way until it hits the sink *t* at which point it disappears. In their classic synthesis, Doyle & Snell [17] provide a thorough discussion of the relationship between such ‘random walks and electric networks’. In this relationship, voltage can be thought of as the probability a particle at node *i* arrives back at the source before arriving at the sink and current is the expected number of particles passing from *i* to *j* minus the number of particles passing from *j* to *i* per unit time.

This random walk is just one of a number of microscopic models that produce the macroscopic observations of current and voltage [18,19]. Kelly [18] gave the following alternative description, which naturally gives rise to a stochastic simulation of a random walk. Consider a graph in which every node is labelled as either occupied by one particle or empty, i.e. with *N_i_* ∈ {0,1}. On each time step of the simulation one edge in the network is chosen at random to be updated. The probability to choose edge *ij* is2.2
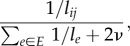
where *E* is (unlike *E_i_* in equation (2.1)) the set of all edges in the graph. The source node *s* becomes occupied at a fixed rate *ν*, which is also equal to the rate at which particles at the sink node *t* disappear. For all other edges, the resistance of an edge gives the rate at which it is updated. The particles on the nodes of the chosen edge are then swapped, so that if *i* is empty and *j* is occupied, then *j* becomes empty and *i* becomes occupied. If both nodes are occupied (or empty), then their status is unaltered, i.e. flow occurs only when one is empty and one is occupied. The mean flow rate on edge *ij* is thus proportional to2.3
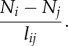
When the system evolves to equilibrium, the probability of node *i* being occupied can be treated as the voltage on the node.

**Figure 2.**
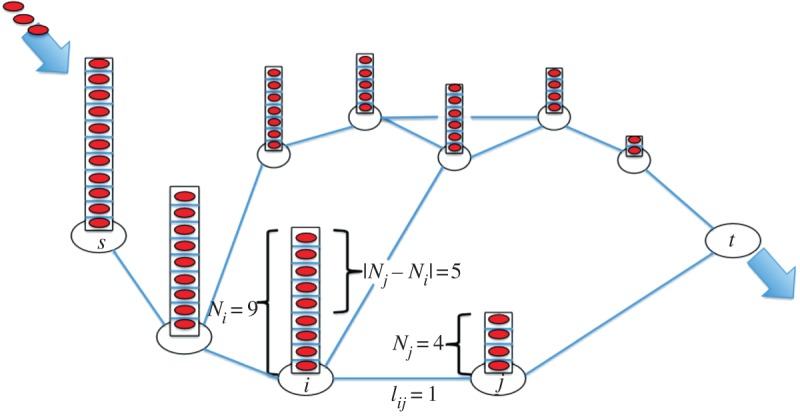
Illustration of the stacked-particle flow model in a network. *s* represents the source where the external particles flow in the network, *t* represents the sink where particles disappear. As an example, in the present state, node *i* has nine particles and node *j* has four, the length of edge *ij* is one, then according to equation (2.4), at present the flow rate through *i* to *j* is 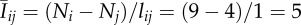. (Online version in colour.)

Figure 2 shows a network with *m* ‘stacked’ networks, with *N_ik_* ∈ {0,1} particles on node *i* in the *k*th network. If we run Kelly's model independently and simultaneously on each of these networks, then the summarized mean flow rate on edge *ij* for each network is2.4
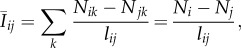
where 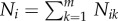 now denotes the total number of particles stacked on node *i*. Because particles move independently, the particles moving along edge *ij* during a small fixed time interval *Δt* approximately follows a Poisson distribution2.5
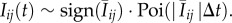

The direction of the movement is determined by the sign of *N_i_* − *N_j_*. If *N_i_* > *N_j_*, then the flow goes from *i* to *j*, otherwise it goes from *j* to *i*, i.e. *I_ij_* =−*I_ji_*. Thus, in each time step, the number of particles on each node is updated as2.6
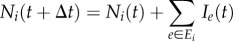
where *E_i_* is the set of edges connected to *i* as in equation (2.1). The source of the network has an input current so that the number of particles at *s* is replenished with a rate, 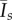, while the sink has an output current rate 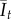. *N_i_*−*N_j_* is the voltage or potential difference and equation (2.4) is equivalent to Ohm's law.

**Figure 3.**
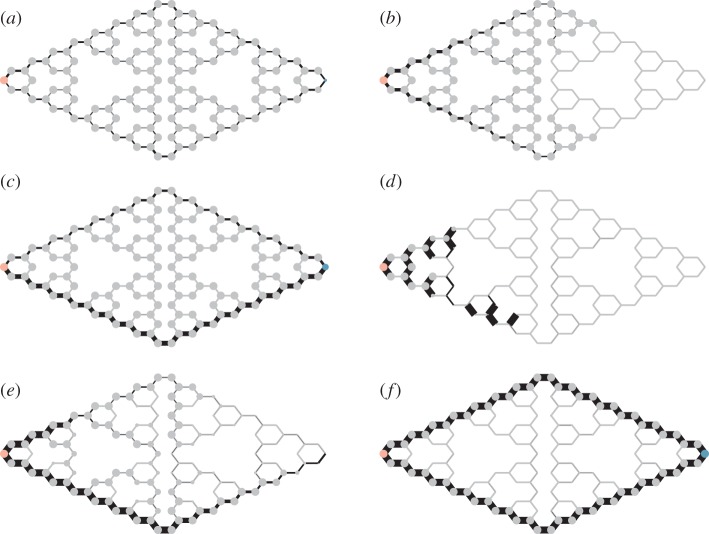
Simulations of different microscopic models on the Tower of Hanoi maze. In each plot, the pink dot on the left end of the maze is the source, and the dot on the right end of the maze is the sink. The thickness of the black line represents the average flow going through the network in the previous time unit. The sizes of the nodes are proportional to the number of particles on each node with an upper truncation. In the simulations, we set external flow rate 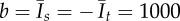 per unit time for all models. (*a*) Random walk, *Δt* = 0.001; (*b*) current-reinforced random walk, near the start of the simulation, *q* = 0.01, *λ* = 1, *Δt* = 0.01, *D*_min_ = 0.001; (*c*) current-reinforced random walk, in equilibrium; (*d*) simple ant colony optimization, *Δt* = 0.001, *D*_min_ = 0.001; (*e*) biologically realistic-reinforced random walk near the start of the simulation, *q* = 0.0001, *λ* = 0.001, *Δt* = 0.1, *D*_min_ = 10^−5^; (*f*) biologically realistic-reinforced random walk, in equilibrium. The Matlab code to generate these results is available at http://www2.math.uu.se/qi/Videos.html.

Figure 3*a* gives an example of the equilibrium state of 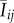 and *N_i_* on the Tower of Hanoi network. The particles do a random walk throughout the network. Flow of particles occurs everywhere on the network, although it is stronger on the edges corresponding to the shortest path.

### 2.2. Current-reinforced random walks

So far we have discussed several microscopic descriptions of electric networks, but in these cases, the particles are not aware of or responding to the voltage and current they are generating. We now keep the microscopic movement of particles as in the last description above, but allow the particles to modify the conductivity of the network they move in. Unlike the previous scheme, where the conductivity for each edge is equal to one through time, now the conductivity of the edges is evolving as the particles move around. Let *D_ij_*(*t*) denote the conductivity of an edge in the network at time *t*, then by Ohm's law the mean flow rate on each edge becomes2.7
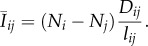


Still, the current of each edge is a Poisson-distributed random number as in equation (2.5). The absolute value of *I_ij_*(*t*) is the number of particles moving along the edge during *Δt*, and the sign of *I_ij_*(*t*) denotes the direction of the moving. The input and output external currents are fixed to 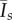 and 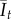 at the source and sink, respectively. Numbers of particles on the nodes are updated the same way as shown in equation (2.6). In terms of ants, *D_ij_* can be thought of pheromone concentration and *N_i_* as number of ants. Table 1 gives interpretations of the model parameters and variables in terms of electrical networks, of ant trails and in terms of the biology of the slime mould *Physarum*.

**Table 1.** Term interpretations for different systems.

parameter name	electric network	*Physarum*	ant trails
*l_ij_*	length in space	length in space	length in space
*N_i_*	potential/voltage	amount of nutrient	number of ants
*I_ij_*	current	flow of nutrient	flow of ants
*D_ij_*	conductivity	thickness of tube	pheromone concentration
*C_i_*	capacitance	transport efficiency	total pheromone density
*q*	reinforcement intensity	tube expansion rate	pheromone drop rate
*λ*	conductivity decrease rate	tube decay rate	evaporation rate

To introduce reinforcement, we update the conductivity *D_ij_* in response to the current after the moving of the particle as2.8
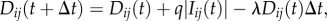
where constant *q* represents the reinforcement intensity caused by per unit flow, and *λ* denotes the decreasing rate for the conductivity. Equation (2.8) implies that at each step the conductivity of each edge decreases slightly, but the edges with current have their conductivity increased. These rules define our *current-reinforced random walk*.

Figure 3*b,c* shows two different time points in the time evolution of the above algorithm on the Towers of Hanoi network. At first, the particles spread over the whole network, and then the shortest path is formed and reinforced. Finally, at equilibrium, the flow converges to the shortest path. In general, simulation of this system on mazes converges to the shortest path. The simulation also responds effectively in a dynamic environment. Electronic supplementary material, movie S1 is an example of the current-reinforced random walk in which the network structure is changed in the same way as the experiment by Reid *et al.* [1] on Argentina ants. Both in the model and the experiment the particles/ants adjust to the shortest path the maze was changed.

Equation (2.8) allows for discrete stochastic dynamics of the conductivity. If we take *Δt* → 0 and ignore stochastic fluctuations, we get2.9
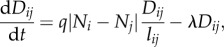
where the first term on the right-hand side is now the expected absolute value of the current rate. This equation is the mean field version of equation (2.8). Importantly, this mean field updating of conductivity is used in the *Physarum* solver model, first described by Tero *et al.* [16]. In the *Physarum* solver, the body of slime mould is assumed to be a network of pipes. In terms of *Physarum* biology, the flow rate of protoplasm can be thought of as 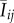 and the thickness of the pipe as *D_ij_*. *q* is the intensity of reinforcement and *λ* the degeneration rate of the pipe, *l_ij_* is the length of a pipe, *N_i_* is the ‘pressure’ at a node. By taking *Δt* → 0 in equation (2.6), the dynamics of the pressure can also be described continuously as2.10
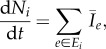
where 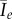 is now the expected flow rate as in equation (2.7).

If *q* and *λ* (in equation (2.9)) are small, then the conductivity *D_ij_* evolves much slower than *N_i_*. In such a situation, *N_i_* and *D_ij_* are, respectively, fast and slow variables allowing time separation between them. *N_i_* can thus be treated as in equilibrium, i.e.
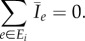


This equation is Kirchhoff's current law, that the flow in to a node equals to the flow out. By solving this equation instantaneously every time after *D_ij_* is updated, one can obtain the corresponding *N_i_* and in advance get the flow rate 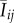. Under this scheme, *D_ij_* is updated as in equation (2.9). This is the simulation scheme used by Tero *et al.* in their original *Physarum* solver [16].

The scheme of Tero *et al.* can allow for a faster numerical solution of the above equations. However, unlike in equation (2.10) and our stochastic model, this scheme is no longer decentralized. The advantage of the stochastic simulation is that to update any one edge we need to know only the flows into that edge, whereas a solution of Kirchhoff's law depends on a global solution over all nodes. This point is important both regard to the biological realism of the model and if the model were to be implemented efficiently on a parallel computer.

### 2.3. Density reinforcement

We now contrast the current-reinforced model with models in which reinforcement is based on density. Based on experimental work and models of ant foraging [20], Dorigo & Stutzle [21] proposed the following reinforced random walk algorithm. In their model, each ant at node *i* walks down an edge *ij* with probability2.11
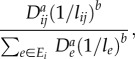
where, in terms of ants, *D_ij_* can be thought of as the concentration of pheromone on edge *ij*. Here, we consider the simplest case *a* = *b* = 1, where the ants use the same resistance rule as in equation (2.1), i.e the probability of an ant moving from *i* to *j* next step is2.12
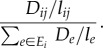


The pheromone concentration *D_ij_* in the ant system is assumed to increase in proportion to the number of ants pass through edge *ij*, i.e. traffic flow of ants on that edge, and decrease with evaporation at rate *λ*. The update rule for *D_ij_* is then the same as in equation (2.8).

Under this scheme, the particle flow in both directions between two nodes are taken into account, i.e. the flow on edge *ij* equals to the number of particles going from *i* to *j* plus the number of particles going from *j* to *i*. The mean flow rate between node *i* and *j* is given by2.13
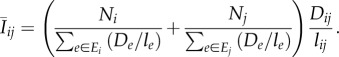


There is clearly a strong analogy between this density-reinforced random walk and the current-reinforced random walk described earlier. However, there are two important differences between the ant system and the *Physarum* solver. First, the pressure/potential is now defined as2.14
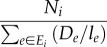
rather than simply being the number of particles. Because *D_ij_* is analogous to pheromone and *l_ij_* is the length of a path, we can think of *D_ij_/l_ij_* as pheromone density. Thus2.15
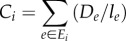
is the total pheromone density going in and out of node *i*. In the sense of electric network, each node now can be considered as a capacitor, and *C_i_* is thus the capacitance. Second, as mentioned earlier, the flow between node *i* and *j* here is a sum of particles moving from *i* to *j* and from *j* to *i*, while in the current reinforcement random walk, particles only move in one direction depending on the sign of *N_i_* − *N_j_*. This difference is reflected by the different sign in equations (2.7) and (2.13).

Reinforcement based on the sum of the pressure on nodes results in a tendency for the ants/particles to stay longer nearer to the source and it hinders them from extending outward to new available space. Figure 3*d* shows a stochastic implementation using equation (2.13). The particles are trapped in their own feedback loop and cannot establish the shortest path between the source and the sink. Dorigo & Stutzle [21] proposed loop-erased random walks to prevent particles getting trapped in their own feedback. In their simulation, they let only one ant at a time go through the maze. This ant does not deposit pheromone until it gets to the sink. Once it has arrived at the sink, all loops in its path are removed, and pheromone is laid on the paths not involving loops. These loop-erased random walks are ultimately equivalent to the random walks based on current reinforcement. This can be seen by noting that loops are equally likely in either direction and thus cancel out, leading back to reinforcement based on potential differences. The advantage of our current-reinforced random walk over loop erasion is that it is based entirely on local information.

### 2.4. Biologically realistic model

Although the current-reinforced random walk in §2.2 finds the shortest path through a maze it should not be considered fully biologically realistic in the case of the ants. In figure 3*c*, we see that the number of particles/ants on the nodes without flow passing by are approximately equal to that on nodes where there is a flow. In biological terms, this is equivalent to ants placing themselves at all positions in the foraging arena, but moving only on the path which is the shortest!

We can improve the biological realism by thinking about the decisions by an individual particle or ant about how to move through the network when responding to the local pheromone density. We first assume that
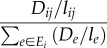
is the probability that an ant on node *i* will chose to move to node *j*. This rule is consistent with how Argentine ants react to pheromone trails, where ants turn towards areas with higher relative pheromone density [22]. The total rate of traffic from *i* to *j* is
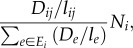
and the flow of traffic between *i* and *j* is then given by Ohm's law as2.16
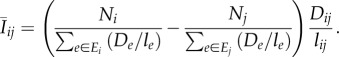


During time interval *Δt*, the flow from *i* to *j* is approximately Poisson distributed, as defined in equation (2.5). The number of particles on each node and the conductivity on each edge is then updated using the same rule as equations (2.6) and (2.8), respectively. Note that equation (2.16) requires information from second-order neighbours in the network. To obtain a neighbour node's total pheromone density, the conductance of all edges linked to this neighbour must also be calculated.

The difference between equations (2.7) and (2.16) is in the introduction of a capacitance, *C_i_* (defined in equation (2.15)). *C_i_* is equivalent to the total pheromone density of node *i* and equation (2.16) states that ants are more likely to move towards nodes that have relatively large total pheromone density. In equation (2.7), the potential was simply the number of particles on the node, i.e. *C_i_* = 1 for all nodes, whereas in the biologically realistic model (equation (2.16)), the number of particles is rescaled by the total pheromone density on the node. As a result, edges linked to nodes with large number of ants can still have flow if the two nodes have different total pheromone density. Larger *C_i_* decreases the potential of the node and the ants will move to a lower potential node and create a flow.

Figure 3*e,f* shows an example of this model solving the Tower of Hanoi maze. Now both the flow and concentration of particles lie on the shortest path, in exactly the same way as they do in both ant and *Physarum* experiments (figure 1). Electronic supplementary material, movie S1 provides an example of the current-reinforced random walk in which the network structure is changed in the same way as the experiment by Reid *et al.* [1]. When the network is changed, both the ants and the flow focus on the shortest path.

### 2.5. Non-symmetric *Physarum* solver

In the earlier-mentioned numerical examples, our current-reinforced random walks converge to the shortest path between a source and a sink. In order to prove the convergence of these mechanisms in a general sense, Johansson and co-workers have proposed a slight modification to the *Physarum* solver in which conductivity is updated in both directions between two linked nodes [23,24]. In this modification, between node *i* and *j*, there are edges in both directions. When *N_i_* > *N_j_*, the flow rate on edge *ij* will be 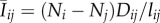, while on the other direction 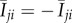. Because 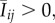 the conductivity will be reinforced on edge *ij*, but at the same time 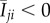 so the conductivity decreases on edge *ji*, thus the conductivity will be updated non-symmetrically. Specifically, the conductivity *D_ij_* has the deterministic dynamics2.17
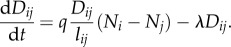


The use of a bidirectional conductivity vector of *D_ij_* takes us away from the electric network analogy, where conductivity and conductance work symmetrically at a fundamental level. A corresponding electric network for this model will have an effective conductivity *D_ij_* + *D_ji_* between node *i* and *j*. The important point however is that of the two edges *ij* and *ji* at most one can be increasing at any one time. If the flow is from *i* to *j*, then the conductivity *D_ji_* in the opposite direction will decrease with a rate *O*(*e*^−*t*^) as *t* → ∞.

Rewriting equation (2.17) in the following form,2.18
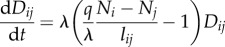
we can see that at equilibrium it gives two possible solutions for the solver, either *D_ij_* = 0 or 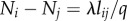 and *D_ij_* > 0. When *D_ij_* = 0, Ohm's law (equation (2.7)) implies that 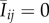 so there is no current between *i* and *j*. This means that at equilibrium, flow moves along only some edges on the graph, then the question is to prove that these edges that have *D_ij_* > 0 are lying on the shortest path. This is done by removing all negative cost cycles in the graph. Details of the proof can be found in Ito *et al.* [23], along with an application to linear programming problems [23,24].

## 3. Transport networks

**Figure 4.**
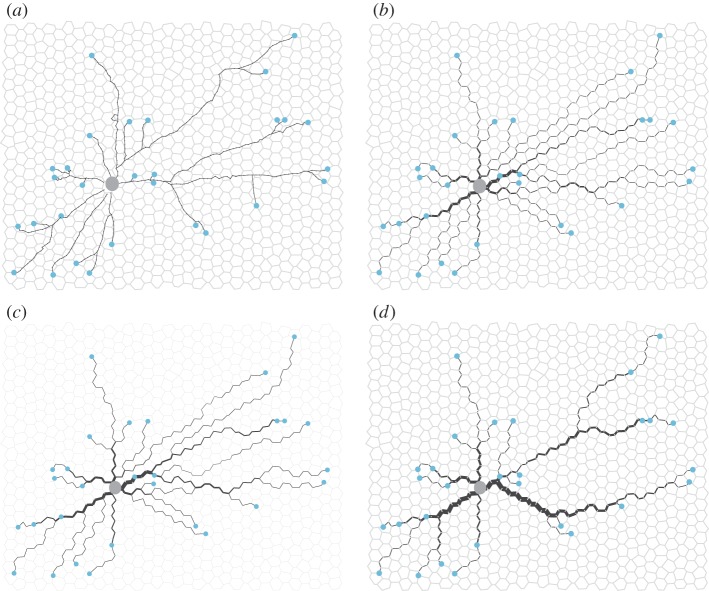
Comparison of wood ant trail and networks formed by implementations of different random walk models using the location of the ant nest as the source and locations of trees as sinks in the simulations. The large grey dot in the centre of each subfigure represents the source where particles enter the system. Small green dots at the extremities of each sub-trail represent sinks where particles are subtracted from the system. The thickness of the black lines on the edges is proportional to the conductivity of the edge. (*a*) Ant trail no. 1; (*b*) network formed by current reinforced random walk; (*c*) network formed by biologically realistic random walk; (*d*) network formed by nonlinear biologically realistic random walk with *μ* = 1.1. Parameters for the simulations: *Δt* = 0.1, *q* = 10^−5^, *λ* = 10^−3^, 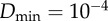, external particles flow-out rate at each sink *b_t_* = 100, flow-in rate is *b_t_* times the number of sinks.

Many biological systems connect multiple source and sinks to form networks. For example, slime moulds link all the food sources in their searching range and form efficient transport networks [6]. Similarly, many ant species have several nests among which the workers, brood and resources are reallocated to improve foraging efficiency and competitive ability [25]. Buhl *et al.* [7] studied networks created by wood ants connecting food sources to a central nest (figure 4*a*). They characterized the networks in terms of two components of efficiency: the route factor and total edge length [26]. The route factor is the average of direct distance between each node and the central node while the total edge length is the sum of all the edges in the measured network. The wood ants' networks consist of a main trunk trails from which branching trails connect nearby food sources, providing both low route factor and low total edge length.

There are some similarities between the reinforced random walk models and the process of ant and slime mould network formation. For example, Latty *et al.* [5] found that in Argentine ants the process of network formation involves an initial construction of multiple links followed by a pruning process that reduces the number of trails. A similar pruning process is seen in foraging army ants, which show an initial expansion followed by a contraction to keep only trails leading to food items [27,28]. Perna *et al.* [29] showed that a similar pruning mechanism is consistent with the structure of chambers and passageways in termite mounds. Slime moulds show a similar network formation process among several food sources [4,6].

To compare our random walk models with these biological systems, we now incorporate multiple sinks. We implement both the current-reinforced random walk (§2.2) and the biologically realistic model (§2.4) on a randomized hexagonal grid with a central source and sinks placed at various places around the grid. To compare with the data of Buhl *et al.*, the source can be thought of as the ant nest and the sinks are located at the positions of the trees at which the ants forage (figure 4*a*). The ants are simulated as particles going out from the source to find the sinks. The current-reinforced random walk model converges to the one-to-one shortest path from the nest to each tree (figure 4*b*). The biologically realistic random walk model also converges to nearly the one-to-one shortest path from the source to each sink, but with a few redundant edges owing to larger random fluctuations (figure 4*c*). The solution produced by these models have the lowest route factor, but they do not provide an efficient network in terms of total edge length. Nor does this solution look like the network built by the ants (figure 4*a*).

To understand why the current-reinforced random walk produces a low route factor network, but not one that looks like a real ant foraging network, we should consider the type of optimization problem which the current-reinforced model solves for multiple sinks. Johansson *et al.* [23] prove that the non-symmetric *Physarum* solver (equation (2.17)) minimizes the following cost function3.1
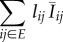
subject to **B*I*** = ***b*** and ***I*** ≥ 0, where **B** is the adjacency matrix of the network, ***I***_m×1_ is the flow vector goes through each edge and ***b***_n×1_ is the external flow on each node. If *b_i_* > 0, then *i* is a source and if *b_i_* < 0 it is a sink. The total inflow equals the total outflow, i.e. 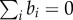. Here, the total cost, i.e. equation (3.1), represents the total power caused by the flow at equilibrium. In electric networks, the power needed to transport one unit flow between two nodes depends on the potential difference/voltage drop *U_ij_*, when Ohm's law is valid, i.e. 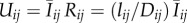. The cost *U* of transporting per unit flow grows linearly with the flow. As a result, when there is one source and multiple sinks the solution is simply a union of the one-to-one shortest paths between every sink and the source, as seen in figure 4*b*.

In many biological systems, the cost of transporting per unit flow does not grow linearly with the flow. For example, for ants, there is a cost associated with maintaining trail and clearing it from debris which decreases if many ants are using the trail [30,31]. In such cases, per unit flow can be modelled by a concave cost function such as3.2
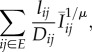
where *μ* > 1. Transportation systems optimized in this way require particles to share the same path near to the source, because the cost of per unit flow is smaller when the flow increases. As a result, the networks formed in these systems have smaller total length than the networks formed by linear cost optimization. Furthermore, unlike the linear cost problem, when *μ* > 1 this problem is NP-hard [32], implying there is no general computational efficient algorithm to construct solutions.

The form of the optimization problem suggests a possible heuristic method for its approximate solution. If we consider the cost per unit flow as the potential difference/voltage drop, then equation (3.2) can be considered to be
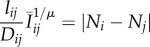


Note the optimization problem is subject to 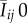, therefore we use the absolute value in the calculation. The corresponding flow rate is then
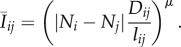
which can be substituted in to equation (2.7), and then used correspondingly in equations (2.5), (2.6) and (2.8) to specify a nonlinear-reinforced random walk.

In the biologically realistic random walk model, the same argument can be applied just with a different pressure/potential. In this case, however, by using this nonlinear flow, Ohm's law is violated. To avoid this problem, we introduce the nonlinearity to the update of the conductivity instead. We set the evolution of the conductivity as3.3
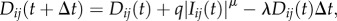
where *I_ij_*(*t*) is defined the same as in equation (2.5). In terms of ants, this rule implies that amount of pheromone deposited by individual ants increases super-linearly with the flow. Figure 4*d* shows the networks formed by this model, i.e. equations (2.16), (2.5), (2.6) and (3.3) when *μ* = 1.1. This nonlinear biologically realistic model gives networks with a high route factor (r_*_ = 0.97 for the simulated network and r* = 0.70 for the real ant network) but low total edge length (L* = 0.89 for the simulated network and L* = 0.84 for the real network) and similar to that of real ant trails (see Buhl *et al.* [7] for a definition of r* and L*). The similarity suggests that nonlinear response of the ant flow to the pheromone density change might be the mechanism used by ants. Tero *et al.* [6] use a mean-field variant of equation (3.3) in modelling *Physarum* network formation, and also drew the conclusion that with nonlinear response of the flow the networks formed resembled those formed by *Physarum*.

While we have used current reinforcement in our simulation, it is density reinforcement which is used in the majority of previous models of biological aggregation and trail formation [10,15,28,33]. To model pattern formations in chemotactic environment, Othmer and Stevens assume that the reinforcement is proportional to the local density of particles and or the control substance (pheromone). These models reproduce general aggregation patterns of particles, but do not form the spatial networks seen in figure 4*d*. Earlier biologically motivated trail formation models also use density reinforcement. For example, Edelstein-Keshet *et al.* assume that follower ants and lost ants lay pheromone at a constant rate, so ant trails are reinforced proportionally to the number of follower ants and lost ants in local area. As a result, and similar to in figure 3*d*, this model gives ant trails with many loops [33]. Other models of ant and human trails, as well as vessel formation, that use density reinforcement, can reproduce more realistic tree-like trail structures. However, these results depend on introducing global navigational information [15], a gradient that biases growth towards one particular direction [34] or by treating outbound and inbound particles differently [28]. The difference between our current-reinforced random walk and these approaches is that our model is based entirely on local information and does not require that particles have memory. As such, the assumptions underlying current reinforcement are consistent with the biology of both slime moulds, ants and other biological systems.

## 4. Conclusion

In the context of foraging by trail-laying ant species, the model presented in this study makes a minimum of assumptions about the individual navigational capabilities of ants. The first of these assumptions is about how ants follow pheromone. Recent experiments have established that Argentine ants turn at a rate proportional to4.1
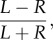
where *L* is the pheromone concentration to their left and *R* is concentration to the right [22]. This rule is consistent with equation (2.16), supporting this basic model assumption about how ants follow pheromone. Different evaporation rates and pheromone sensitivity for different species determine the parameters *λ* and *q*, respectively. However, in the model, the eventual establishment of a shortest path does not depend crucially on these parameters. Instead, these will determine how quickly ants will react to changes in their environment.

The second model assumption is that ants modify their trail-laying behaviour with the flow of ants on a trail. Such regulation has not as yet been tested experimentally. Ants of most trail-laying species pause to interact with nestmates on the trail, providing a possible mechanism for regulation [35]. The prediction of our model is that ants lay less pheromone when they encounter a flow of ants moving in the other direction. For example, one behaviour consistent with the model is that ants leave pheromone whenever they find food, but when these fed ants meet other fed ants moving in the opposite direction then they reduce their tendency to lay pheromone. In cases where ants leave pheromone both before and after they find food, their trail-laying tendency should reduce if they encounter ants in the same state (fed/unfed) as themselves moving in the opposite direction. Testing these assumptions requires experiments in which the interactions of ants on the trail are observed and the effect these have on trail-laying propensity measured.

A third additional assumption is made in §3 in order to generate transport networks. Here, we include a nonlinear interaction between traffic flow and pheromone deposition. This nonlinearity is not captured directly in equation (4.1), but there is evidence of similar nonlinearities in interactions between ants, pheromones and their environment [20,22]. We would expect the exact details of interactions to vary from species to species, providing different values for the nonlinearity parameter *μ*, as it does with the pheromone parameters *λ* and *q*. Moreover, many species of ants exhibit more complex behaviour during foraging than are included in our model. Some species leave multiple pheromones depending on whether they are exploring or they have found food [36]. Other species use environmental navigational cues, such as path integration and compass [37]. Earlier models of the foraging ants have shown that including these extra capabilities, together with density reinforcement via pheromone, produce somewhat realistic network structures [14,28]. We have shown that even in the absence of these additional cues, ants can use current reinforcement to build foraging networks.

The current-reinforced random walk description provides biological underpinnings to the *Physarum* model proposed by Tero *et al.* [16]. The *Physarum* model is described by a set of ordinary differential equations on a network, which we have shown to be a particular deterministic limit of the current-reinforced random walk in §2.2. One important difference is that while random walk models are local, the *Physarum* solver uses global information in calculating Kirchhoff's law on each time step. Another difference is that the *Physarum* model does not explicitly deal with the problem of having large numbers of particles in areas of the network where there is low flow. These differences are important if our aim is to describe the decentralized construction of transport networks.

We expect current reinforcement to be important in a much wider range of biological systems than ant foraging. Indeed, at the microscopic level, cells and other biological ‘particles’ do not have any additional navigational capabilities. Work on reinforced random walks in, for example, tumour angiogenesis, has used density-reinforced random walks combined with a gradient bias to model chemotaxis cell migration, leading to blood vessel growth towards a tumour [11,34]. While external gradients may well exist in these systems, we would suggest that current, rather than density, reinforcement is a more realistic paradigm for this type of network formation or morphology problems. Many of these systems involve the flow of biological matter and produce bifurcating networks reminiscent of figure 4*d* [38]. By adopting current reinforcement, these systems can avoid the formation of self-reinforcing loops. Gradients can be represented in our models simply by changing the resistance *l_ij_* of the edges of the network. A similar argument for the application of current reinforcement can be made for neural networks, where electrical signals created by chemicals reinforces the connections between neurons [39–41]. We expect the biological details of each system to be different, but the principle of reinforcement where flow and capacitance are large should be common to many of these systems.
